# Supplementary material for: Changes in the VOC of Fruits at Different Refrigeration Stages of ‘Ruixue’ and the Participation of Carboxylesterase MdCXE20 in the Catabolism of Volatile Esters
Source: Foods. 2023 May 12;12(10):1977. doi: 10.3390/foods12101977 (PMC10217609; doi:10.3390/foods12101977)
Supplement: Supplementary file 1 [file foods-12-01977-s001.zip › foods-2327558-supplementary.pdf]

Supplementary Material

Table S1. Schedule Change of volatile substance content during storage period of ‘Ruixue' apples (μg/kg)

Compound	Content（μg/kg）					
	0 d	30 d	60 d	90 d	120 d	150 d
Butanoic acid, propyl ester	-	37.641±2.75	169.848±10.45	106.734±8.09	122.217±8.34	181.196±6.35
Butanoic acid, 2-methyl-, propyl ester	12.384±0.53	17.508±1.05	45.741±3.89	24.084±2.12	24.531±2.45	35.298±2.67
Butanoic acid, butyl ester	39.408±1.98	199.765±9.89	649.924±30.56	297.675±16.89	432.294±20.24	497.244±25.13
Butanoic acid, 2-methylbutyl ester	22.822±1.34	53.804±2.19	95.473±4.45	54.521±1.94	77.201±5.23	69.996±4.37
Acetic acid, hexyl ester	13.905±0.56	74.378±2.75	257.919±10.72	91.221±5.19	22.811±1.93	54.044±4.37
Butanoic acid, 2-methyl-, 2-methylbutyl ester	61.778±4.95	59.348±2.56	58.430±2.87	31.726±3.22	27.002±1.05	33.146±1.64
Butanoic acid, pentyl ester	17.588±0.56	69.941±3.82	144.401±10.30	84.155±4.74	125.896±9.04	160.138±12.05
Butanoic acid, 2-methyl-, pentyl ester	74.402±5.90	90.778±6.07	99.04±4.96	68.079±2.89	33.673±0.45	80.405±2.72
Propanoic acid, hexyl ester	91.156±6.16	144.670±9.11	476.165±45.09	300.508±23.18	208.574±15.21	263.932±17.38
Hexanoic acid, butyl ester	37.100±2.67	472.599±18.02	790.549±59.40	626.144±42.61	489.860±39.02	-
Butanoic acid, hexyl ester	172.956±10.41	964.571±79.90	1697.36±104.4	1571.19±102.5	1811.85±99.34	3033.50±210.2
Butanoic acid, 2-methyl-, hexyl ester	1230.16±98.94	2139.61±156.5	2361.48±112.7	2650.63±145.8	2047.45±134.2	3269.33±211.9
Hexanoic acid, 2-methylbutyl ester	25.740±1.78	112.423±10.13	194.504±12.45	-	-	-
Hexanoic acid, pentyl ester	-	161.167±10.17	245.505±5.94	-	262.17±16.27	-
Hexanoic acid, hexyl ester	130.478±9.06	1140.33±67.90	1602.41±92.91	358.557±29.45	2058.35±153.9	462.706±27.81
Hexyl tiglate	52.665±3.19	10.823±0.78	39.226±1.70	77.418±5.39	66.459±5.41	108.992±29.08
Octanoic acid, hexyl ester	-	36.419±1.41	55.455±4.18	121.261±10.12	82.820±5.06	128.180±8.23
Butanoic acid, ethyl ester	-	-	35.690±3.75	25.546±1.49	31.015±1.58	51.056±5.03
Propanoic acid, butyl ester	-	-	92.674±4.29	28.561±2.16	20.694±4.28	24.695±1.95
Propanoic acid, hexyl ester	-	27.170±1.74	-	-	-	-
Propanoic acid, pentyl ester	-	13.638±0.83	53.644±3.47	-	-	-
Butanoic acid, 2-methylpropyl ester	-	-	-	-	19.755±1.05	23.329± 0.95
Hexanoic acid, propyl	-	-	73.457±2.75	66.754±6.64	69.340±4.38	116.089±10.9
6-Octen-1-ol, 3,7-dimethyl-, formate	-	20.458±0.73	35.590±2.96	31.278±1.28	-	-

Compound	Content（μg/kg）

	0 d	30 d	60 d	90 d	120 d	150 d
Hexadecane, 1,1-bis(dodecyloxy)-	-	-	23.433±0.69	-	-	-
Hexanal	106.327±5.62	268.722±10.73	713.606±57.81	483.448±39.20	620.856±48.88	793.972±66.31
2-Hexenal, (E)-	-	38.902±1.51	66.199±3.36	-	31.822±3.09	27.121±1.72
2-Hexenal	1022.57±78.19	1570.15±139.9	2553.39±185.3	1423.94±109.9	1432.23±103.3	1303.29±99.41
Octanal	-	-	19.185±0.94	174.30±13.56	202.631±19.58	137.563±13.31
2-Heptenal, (Z)-	-	28.503±2.05	36.425±2.19	34.972±3.08	22.061±1.07	-
Nonanal	56.753±5.48	54.291±4.93	151.055±55.04	36.867±2.27	55.073±3.88	44.798±2.75
2-Octenal, (E)-	20.442±0.79	34.678±1.20	61.586±4.17	43.260±3.61	45.861±3.36	24.010±1.38
2,4-Heptadienal, (E,E)-	-	23.660±0.54	-	-	-	-
Decanal	39.688±2.67	44.529±3.27	118.928±69.41	54.168±3.69	50.501±3.48	-
2-Nonenal, (Z)-	24.379±0.62	63.101±3.55	106.472±68.11	55.941±4.01	68.254±6.17	92.525±5.99
1-Butanol, 2-methyl-	-	103.433±9.43	251.317±21.39	295.571±21.14	193.477±16.74	251.074±16.15
1-Pentanol	-	-	23.216±0.47	33.259±12.21	24.754±15.49	30.755±3.99
2-Heptenal, (Z)-	40.829±2.11	382.891±12.67	781.407±45.85	996.576±33.72	936.723±58.83	1093.42±99.31
13-Heptadecyn-1-ol	11.942±0.69	-	20.435±0.57	-	-	-
5-Hepten-2-ol, 6-methyl-	-	-	-	260.248±19.88	155.724±13.11	450.005±39.98
1-Butanol	-	82.988±65.03	208.871±11.23	239.291±11.59	142.188±11.84	184.115±8.41
1-Octen-3-one	12.164±0.99	11.889±0.34	39.745±1.27	18.338±0.23	16.871±0.64	15.414±0.34
5-Hepten-2-one, 6-methyl-	22.796±1.91	139.391±8.10	180.899±1.14	329.364±14.62	298.578±26.33	119.949±9.37
Tetradecane	-	11.289±0.44	-	-	-	17.206±0.58
p-Xylene	12.268±0.26	37.326±1.55	-	-	51.977±	16.365±
cis-á-Farnesene	15.868±0.82	98.499±4.99	59.267±2.01	86.118±5.34	81.745±7.19	66.770±4.74
trans-à-Bergamotene	388.427±29.35	541.361±36.11	1301.80±87.59	269.162±19.19	2051.86±176.7	-
à-Farnesene	914.003±59.37	8219.12±452.5	2563.11±115.1	613.205±49.33	2283.07±167.3	10303.4±893
Estragole	19.868±0.36		31.899±1.33	-	-	-
cis-Thujopsene	18.987±1.87	-	72.968±4.59	126.281±9.79	89.621±6.40	92.208±8.19
Butanoic acid, 2-methyl-, heptyl ester	-	14.609±0.94	19.642±0.96	19.071±1.18	22.349±1.54	37.618±2.66

Note: Values are means ± standard error of three biological replicates; - no such substance has been detected.

Table S2. Predicted properties of different carboxylesterases characterized in plants and of the intracellular locus of the proteins on websites.

Gene name	Gene name	Chromosome	Size/aa	Length of CDS/kp	Subcellular localization	*pI*	Protein W
MdCXE3	MD02G1275900	2	1011	698	cytoplasm	5.04	83705.47
MdCXE5	MD02G1276600	2	1002	656	cytoplasm	5.05	83055.26
MdCXE6	MD03G1273300	3	1041	560	cytoplasm	5.04	85577.45
MdCXE9	MD05G1076400	5	975	782	cytoplasm	5.08	80633.09
MdCXE10	MD05G1078900	5	999	680	cytoplasm	5.08	82941.45
MdCXE12	MD05G1191100	5	960	588	endoplasmic reticulum	5.08	78325.40
MdCXE17	MD08G1226300	8	1094	552	endoplasmic reticulum	5.02	91200.72
MdCXE20	MD10G1068500	10	1023	549	cytoplasm	5.01	85350.25
MdCXE23	MD10G1091200	10	1116	560	endoplasmic reticulum	5.05	92593.40
MdCXE25	MD10G1091900	10	939	527	endoplasmic reticulum	5.08	77709.78

Table S3. Apple genome names corresponding to 42 MdCXE members

Name	Apple ID	Name	Apple ID	Name	Apple ID
*MdCXE*1	MD02G1198400	*MdCXE2*	MD02G1198500	*MdCXE3*	MD02G1275900
MdCXE4	MD02G1276300	*MdCXE5*	MD02G1276600	*MdCXE6*	MD03G1273300
MdCXE7	MD04G1212400	*MdCXE8*	MD05G1060700	*MdCXE9*	MD05G1076400
MdCXE10	MD05G1078900	*MdCXE11*	MD05G1191000	*MdCXE12*	MD05G1191100
MdCXE13	MD06G1052300	*MdCXE14*	MD07G1043400	*MdCXE15*	MD07G1046000
MdCXE16	MD08G1104000	*MdCXE17*	MD08G1226300	*MdCXE18*	MD09G1017600
MdCXE19	MD09G1086100	*MdCXE20*	MD10G1068500	*MdCXE21*	MD10G1091000
MdCXE22	MD10G1091100	*MdCXE23*	MD10G1091200	*MdCXE24*	MD10G1091600
MdCXE25	MD10G1091900	*MdCXE26*	MD11G1176900	*MdCXE27*	MD11G1191000
MdCXE28	MD11G1296000	*MdCXE29*	MD12G1046500	*MdCXE30*	MD12G1227200
MdCXE31	MD13G1108800	*MdCXE32*	MD13G1187400	*MdCXE33*	MD13G1187500
MdCXE34	MD13G1187900	*MdCXE35*	MD14G1045500	*MdCXE36*	MD15G1085200
MdCXE37	MD15G1124600	*MdCXE38*	MD16G1045600	*MdCXE39*	MD16G1188100
MdCXE40	MD16G1188500	*MdCXE41*	MD17G1018400	*MdCXE42*	MD00G1203100

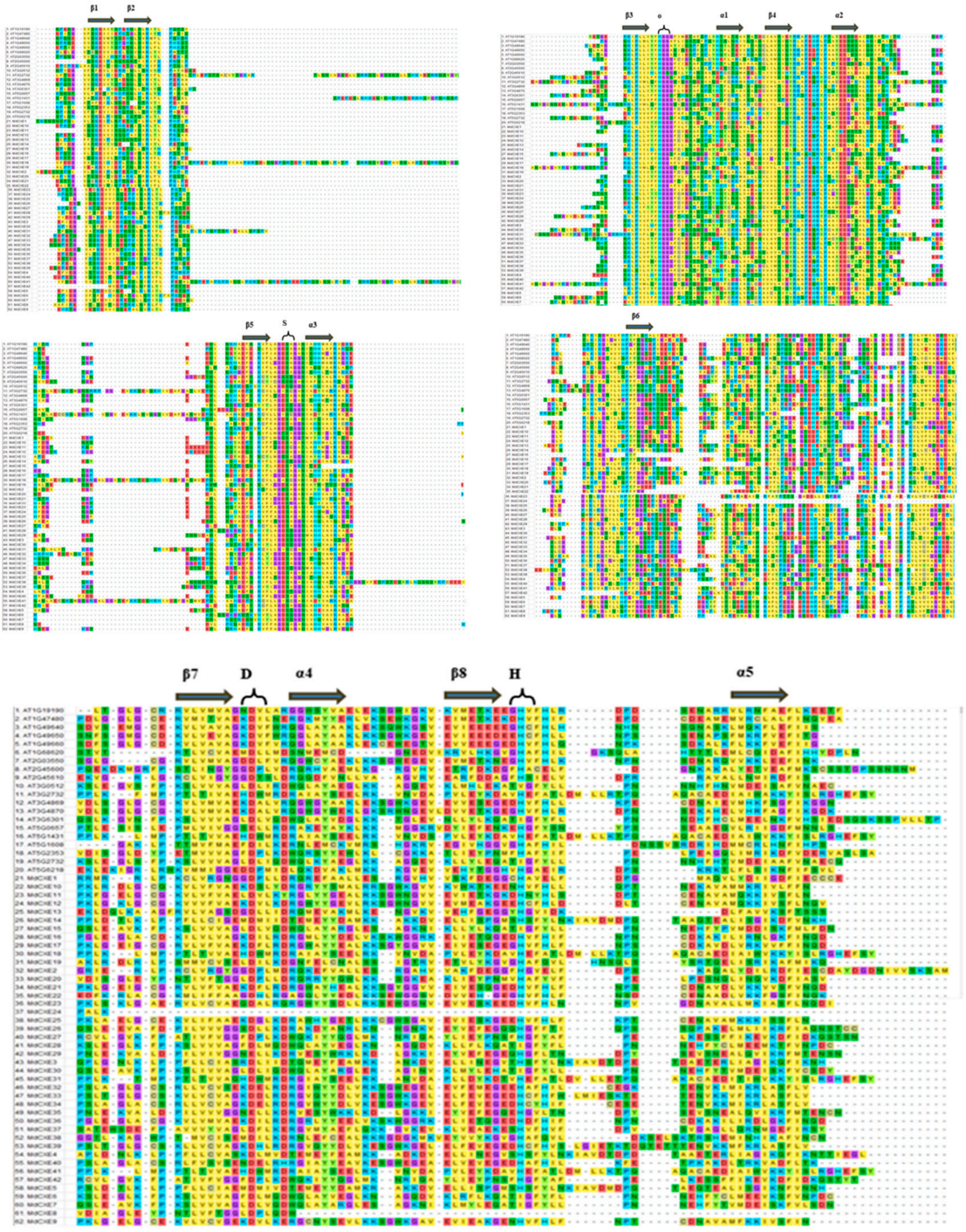

Figure S1. Multiple sequence alignment of CXE gene family in apple and *Arabidopsis thaliana*. The *MdCXEs* α/β hydrolase predicted domain is highlighted in(➡). Putative residues constituting the catalytic triad (Ser/Thr, Asp, and His) are denoted is marked.
